# Pain localization and response to botulinum toxin in cervical dystonia

**DOI:** 10.3389/dyst.2025.14652

**Published:** 2025-07-09

**Authors:** Alexander S. Wang, Hanieh Agharazi, Aetan Parmar, Camilla W. Kilbane, Lauren Cameron, Aasef G. Shaikh, Steven A. Gunzler

**Affiliations:** 1 Movement Disorders Center, Department of Neurology, University Hospitals Cleveland Medical Center, Cleveland, OH, United States; 2 Department of Neurology, Case Western Reserve University, Cleveland, OH, United States; 3 Department of Neurology, Louis Stokes VA Medical Center, Cleveland, OH, United States; 4 Ohio State University, Columbus, OH, United States

**Keywords:** cervical dystonia, pain, pain localization, botulinum toxin, non-motor symptom

## Abstract

**Introduction:**

Pain is a common symptom of cervical dystonia (CD). The mainstay of treatment of CD is botulinum toxin, which is known to have benefits in relieving pain. We aimed to characterize the locations of pain in patients with CD, and to assess what factors may predict pain reduction following botulinum toxin injection.

**Methods:**

We conducted a single-center observational study of CD patients who reported pain and who received botulinum toxin treatment. On the day of their toxin injection (in the untreated state), they filled out a survey evaluating primary and secondary sites of pain as indicated on a diagram, as well as Pain Numeric Rating Scale assessing average pain over the past 24 h. Two weeks later, they filled out a follow-up survey (in the treated state) to evaluate whether location and pain intensity changed.

**Results:**

55 people with CD participated in the study, and 40 of them completed both surveys. Most patients reported pain localization over the posterior musculature, especially in the areas overlying superior trapezius and levator scapulae. 21 of 40 (52.5%) patients reported improvement of pain intensity by ≥ 30% in the primary site of pain. The mean improvement in pain intensity was 30.4% (SD = 32.4%), with a mean improvement on Numeric Rating Scale of 2.13 (SD = 2.02). 68% of patients received injections into or close to their primary site of pain. Using univariate linear regression, there was no clear effect of age, sex, muscles injected, or TWSTRS motor subscale on the degree of pain improvement. The locations of pain remained relatively stable in the post-treatment state.

**Conclusion:**

We confirmed that botulinum toxin is effective for treatment of pain related to CD. We also gained insight into the typical locations of pain in CD by generating a heat map, showing pain most often in the regions of upper trapezius, levator scapulae, and splenius cervicus and capitis. Although there was not a significant correlation between the site of botulinum toxin injection and pain improvement, larger studies are needed to better determine optimal treatment strategies.

## Introduction

Cervical dystonia (CD) is a disorder characterized by involuntary neck muscle contractions causing abnormal postures and sometimes tremor of the head. Compared to other dystonia syndromes, CD patients experience the highest rates of pain [1, 2]. Approximately three of every four CD patients experience pain, which is often described as pulling, aching, deep, or sharp [2-5]. The pain tends to be unilateral involving the muscles near the upper trapezius, but often radiates down one arm or higher up towards the occiput [4].

Botulinum neurotoxin (BoNT) is the most common and most effective treatment of choice for CD [6, 7]. The benefits of BoNT treatment on motor responses, quality of life, and pain have been well established, and many studies have demonstrated that a majority of patients report some degree of pain relief [8-10]. Although botulinum toxin has demonstrated clear benefit, a large portion of treated patients continue to report incomplete satisfaction, often owing to inefficacy, side effects, or logistical reasons [11].

The objectives of our study were first to characterize the typical locations of pain in patients with CD, as well as to assess the effects of botulinum toxin on the locations and severity of pain. Next, we aimed to determine what clinical and demographic factors predicted pain improvement. We hypothesized that patients who received higher doses of toxin, and who had injections directly into their muscular location of pain, would have superior pain improvement.

## Materials and methods

### Bioethics statement

The study involving human participants was reviewed and approved by University Hospitals Institutional Review Board. The participants provided written informed consent to participate in this study. Written informed consent was obtained from the individual for the publication of the identifiable diagram photo included in this article.

### Study design

**FIGURE 1:**
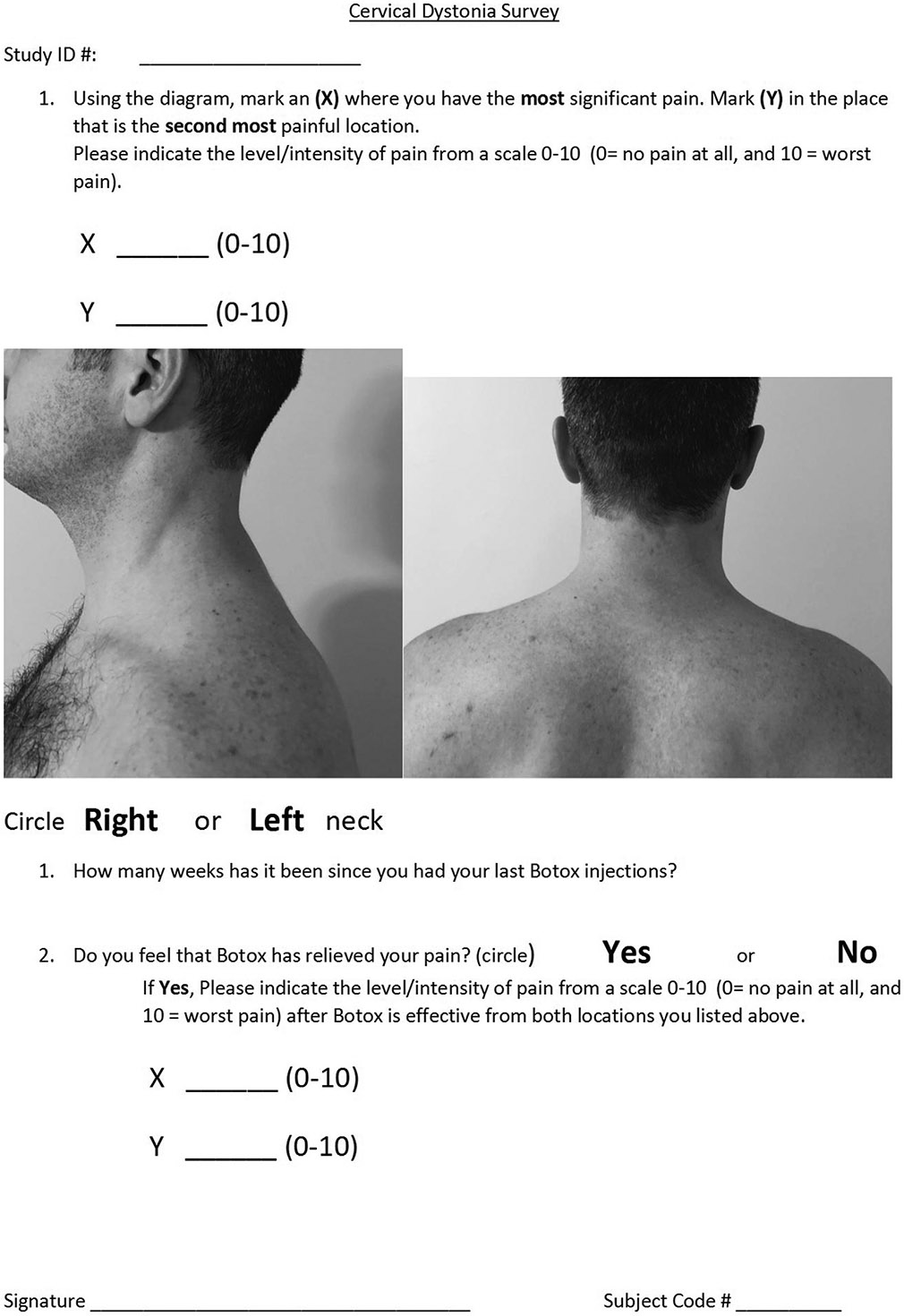
Survey provided to CD patients on the day of their BoNT injection as well as 2 weeks later to evaluate patterns of pain in the untreated and treated state.

We conducted a single-center study of idiopathic CD patients who reported pain and who received botulinum toxin treatment. Patients were recruited on the day of their BoNT injection appointment at University Hospitals Cleveland Medical Center in Cleveland, OH, USA. The patients all received electromyography-guided BoNT injections from movement disorders neurologists and if possible, also underwent clinical evaluation of CD severity using the Toronto Western Spasmodic Torticollis Rating Scale (TWSTRS) motor subscale. On the day of the botulinum toxin injection (in the untreated state), patients filled out a survey evaluating primary (most severe) and secondary (second most severe) sites of pain as indicated on a diagram, as well as Pain Numeric Rating Scale assessing average pain over the past 24 h (Figure 1). The patients were then given a follow-up survey 2 weeks later (in the treated state) to re-evaluate whether location and pain intensity changed.

A virtual adaptation of the same survey, in addition to a TWSTRS examination conducted over video visit, was also given to some patients for whom there was a time constraint against completing the in-person evaluation.

Patients who did not submit a second survey or who filled out an invalid or incomplete response were excluded from that part of the analysis.

### Statistical analysis

We calculated the distance between pain locations, comparing the pre-treatment survey and post-treatment survey by overlaying the diagram responses onto a digital grid allowing standardized pixel distance measurements.

We created a heatmap of combined survey responses and collaborated with a medical illustrator to draw in the expected locations of underlying muscles. We then used this composite diagram along with the clinical documentation to identify whether the patient’s site of pain was injected directly with BoNT to one of the overlying muscles. For instance, if the marked site of pain overlapped with both splenius cervicus and splenius capitis on the overlaid diagram, then injection into either muscle would count as injection into their site of pain.

We used univariate linear regression to evaluate the contributions of age, gender, disease duration, total BoNT dose, BoNT dose into specific muscles, pain distance between pre-treatment and post-treatment states, TWSTRS motor total score, and TWSTRS motor subscores. We excluded those patients who received abobotulinumtoxinA in the BoNT dose analysis, as the vast majority of patients received onabotulinumA, and there is no well accepted conversion factor between the two toxins. We used the Wilcoxon rank-sum test to compare pain improvement responses between those patients who did and those who did not have injections into their primary site of pain. All statistical analyses were performed with Matlab R2022 using an alpha of 0.05.

## Results

We recruited a total of 55 patients who filled out the first survey, of which 21 were performed virtually and 34 were in person. 40 patients submitted a second survey approximately 2 weeks after the first survey. Baseline characteristics are listed in Table 1.

**FIGURE 2:**
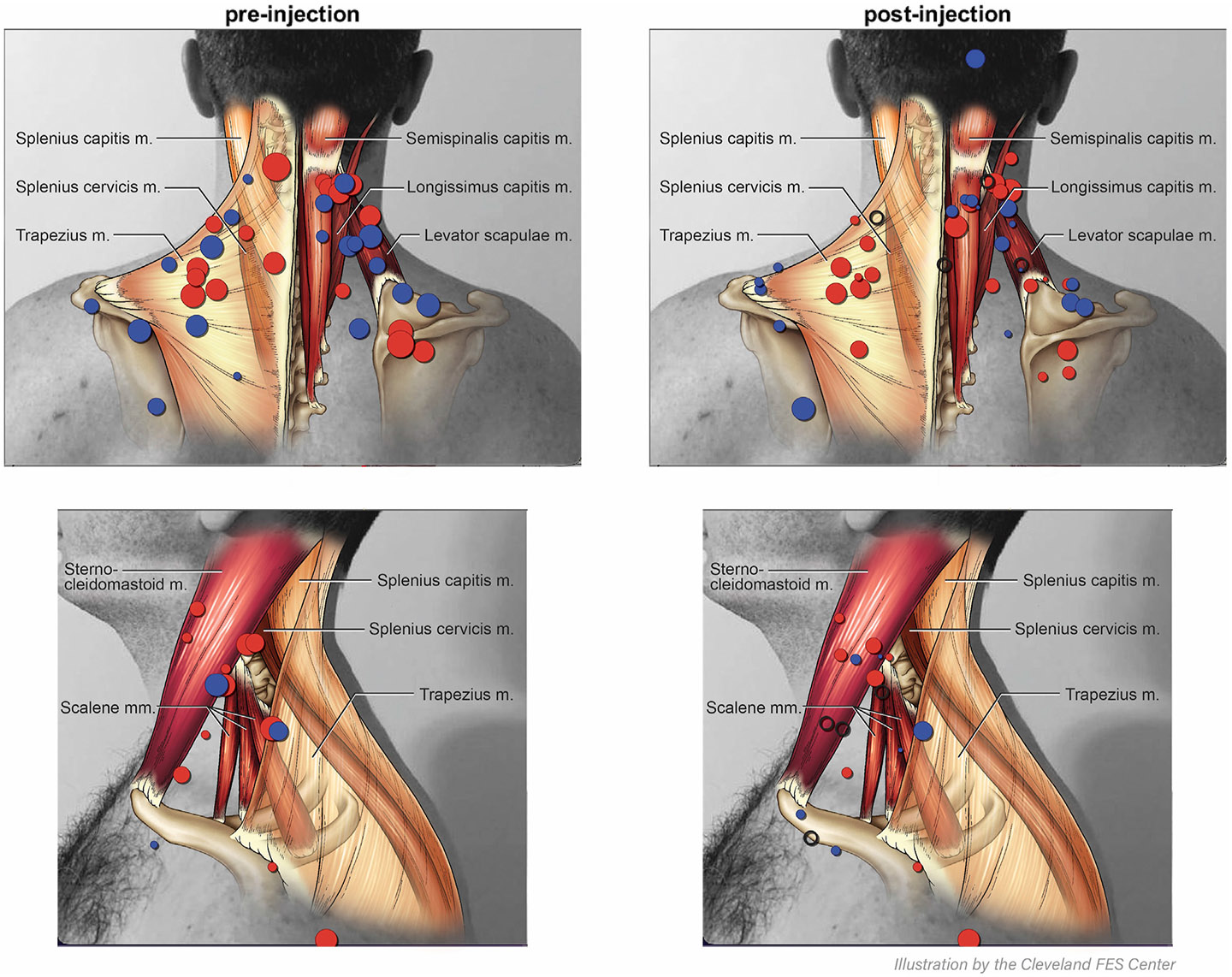
A heatmap of reported primary and secondary pain locations in the treated and untreated states. Red circles indicate primary pain site. Blue circles indicate secondary pain site. The size of the circle is proportional to the degree of reported pain on a scale from 1 to 10. Open circles represent post-treatment areas of resolved pain (reported as 0 out of 10 on the second survey).

31 out of 37 (84%) respondents recorded “yes” that their pain improved following BoNT injection. We did not find any reports of side effects related to BoNT treatment. The mean improvement in pain intensity was 30.4% (SD 32.4%), with a mean (SD) improvement on Numeric Rating Scale of 2.13 (2.02). Those who reported pain benefit had a mean (SD) improvement of 2.44 points (2.19) at the primary pain site on the visual analog scale in the treated compared to untreated state. 21 of 40 (52.5%) patients reported improvement of pain intensity by ≥30% in the primary site of pain. 5 of 40 (12.5%) respondents reported complete resolution of pain. The mean distance between primary pain sites from the baseline to follow-up survey was 70 pixels (SD 81), which correlates with approximately 5 cm. A heatmap of reported pain locations before and after treatment is shown on Figure 2.

There was no clear effect of age, gender, disease duration, total BoNT dose, BoNT doses in individual muscles, TWSTRS motor subscale, or change in pain location on the degree of numerical pain improvement (Supplementary Table S1).

Overall, 33 of 53 (62%) respondents had BoNT injections into their primary site of pain, and 21 of 33 (64%) respondents into their secondary site of pain. The mean (SD) improvement of pain in respondents who had injections into their primary or secondary site of pain (n = 30) was 2.33 (2.4), in contrast to those who did not have injections into either site (n = 9), who reported a mean (SD) pain improvement of 1.33 (2.29) points. There was no significant difference between these two groups (p = 0.30).

## Discussion

We found similar rates of pain improvement in CD following BoNT compared to the existing literature [8]. Our heatmap suggests that the majority of reported pain sites are located around the posterior musculature near the areas of upper trapezius, levator scapulae, and splenius cervicus and capitis. To a lesser degree, anterolateral neck pain was also reported, especially in the region around sternocleidomastoid. The reported locations of pain did not vary significantly between treated and untreated states. Although we did not find any clear predictors of pain improvement, our study was not powered to sufficiently assess this. More study in larger samples may also clarify whether clinicians ought to pursue a “chase the pain” approach in those patients for whom pain relief is a priority.

Notably, there was a discrepancy in perceived benefit as compared to change in numerical pain ratings. Of those six respondents who reported no improvement in pain following injection, three patients wrote a lower numerical pain value on the followup survey. Conversely, 5 of the 31 patients who did report pain improvement wrote either no change or increased numerical pain values on the followup survey. This underscores the contribution of recall bias, as well as a limitation in assessing onetime pain levels as opposed to using more detailed daily pain logs.

One of the main contributions of pain in CD is likely to be muscular in origin, as suggested by typical patterns of pain correlating with muscles that are clinically overactive [4]. However, other lines of evidence have implicated central or multifactorial mechanisms towards pain generation [12-14]. Further studies with more patients and perhaps more detailed and validated pain scales (such as the Pain in Dystonia Scale) are needed to further delineate what factors may predict pain improvement following BoNT [15].

## Supplementary Material

Table 1

The Supplementary Material for this article can be found online at: https://www.frontierspartnerships.org/articles/10.3389/dyst.2025.14652/full#supplementary-material

## Figures and Tables

**TABLE 1:** Baseline characteristics of CD patients on the day of their BoNT injection.

Patient Characteristics	Mean ± SD
Age*	63 years ± 15
Gender*	71% (39/55) Female
Disease duration+	11.0 years ± 9.7
Total Onabotulinum toxin dose#	254 units ± 86
Primary site pain level (1–10)*	6.0 ± 2.4
TWSTRS motor subscale score#	11.7 ± 4.2

* N = 55 subjects

# N = 46 subjects

+ N = 28 subjects.

## Data Availability

The raw data supporting the conclusions of this article will be made available by the authors, without undue reservation.

## References

[1] Hamami F, Baumer T. Pain and cervical dystonia. Schmerz (2024) 38(1):41–7. doi:10.1007/s00482-024-00790-5 38265520

[2] Chan J, Brin MF, Fahn S. Idiopathic cervical dystonia: clinical characteristics. Mov Disord (1991) 6(2):119–26. doi:10.1002/mds.870060206 2057004

[3] Charles PD, Adler CH, Stacy M, Comella C, Jankovic J, Manack Adams A, Cervical dystonia and pain: characteristics and treatment patterns from CD PROBE (cervical dystonia patient registry for observation of OnabotulinumtoxinA efficacy). J Neurol (2014) 261(7):1309–19. doi:10.1007/s00415-014-7343-6 24752807 PMC4098041

[4] Kutvonen O, Dastidar P, Nurmikko T. Pain in spasmodic torticollis. Pain (1997) 69(3):279–86. doi:10.1016/S0304-3959(96)03296-4 9085302

[5] Rosales RL, Cuffe L, Regnault B, Trosch RM. Pain in cervical dystonia: mechanisms, assessment and treatment. Expert Rev Neurother (2021) 21(10):1125–34. doi:10.1080/14737175.2021.1984230 34569398

[6] Rodrigues FB, Duarte GS, Castelao M, Marques RE, Ferreira J, Sampaio C, Botulinum toxin type A versus anticholinergics for cervical dystonia. Cochrane Database Syst Rev (2021) 4(4):CD004312. doi:10.1002/14651858.CD004312.pub3 33852744 PMC8092669

[7] Castelao M, Marques RE, Duarte GS, Rodrigues FB, Ferreira J, Sampaio C, Botulinum toxin type A therapy for cervical dystonia. Cochrane Database Syst Rev (2017) 12(12):CD003633. doi:10.1002/14651858.CD003633.pub3 29230798 PMC6486222

[8] Camargo CH, Cattai L, Teive HA. Pain relief in cervical dystonia with botulinum toxin treatment. Toxins (Basel). (2015) 7(6):2321–35. doi:10.3390/toxins7062321 26110508 PMC4488705

[9] Hefter H, Benecke R, Erbguth F, Jost W, Reichel G, Wissel J. An open-label cohort study of the improvement of quality of life and pain in *de novo* cervical dystonia patients after injections with 500 U botulinum toxin A (Dysport). BMJ Open (2013) 3(4):e001853. doi:10.1136/bmjopen-2012-001853 PMC3641454 23604344

[10] Wissel J, Kanovsky P, Ruzicka E, Bares M, Hortova H, Streitova H, Efficacy and safety of a standardised 500 unit dose of Dysport (clostridium botulinum toxin type A haemaglutinin complex) in a heterogeneous cervical dystonia population: results of a prospective, multicentre, randomised, doubleblind, placebo-controlled, parallel group study. J Neurol (2001) 248(12):1073–8. doi:10.1007/s004150170028 12013585

[11] Comella C, Bhatia K. An international survey of patients with cervical dystonia. J Neurol (2015) 262(4):837–48. doi:10.1007/s00415-014-7586-2 25605434 PMC4544552

[12] Kulisevsky J, Lleo A, Gironell A, Molet J, Pascual-Sedano B, Pares P. Bilateral pallidal stimulation for cervical dystonia: dissociated pain and motor improvement. Neurology (2000) 55(11):1754–5. doi:10.1212/wnl.55.11.1754 11113243

[13] Tinazzi M, Squintani GM, Bhatia KP, Segatti A, Donato F, Valeriani M, Pain in cervical dystonia: evidence of abnormal inhibitory control. Parkinsonism Relat Disord (2019) 65:252–5. doi:10.1016/j.parkreldis.2019.06.009 31227336

[14] Marciniec M, Szczepanska-Szerej A, Popek-Marciniec S, Rejdak K. Pain incidence in cervical dystonia is determined by the disease phenotype. J Clin Neurosci (2020) 79:133–6. doi:10.1016/j.jocn.2020.07.069 33070882

[15] Bruno V, Achen B, Morgante F, Erro R, Fox SH, Edwards MJ, The pain in dystonia scale (PIDS)-Development and validation in cervical dystonia. Mov Disord (2023) 38(7):1175–86. doi:10.1002/mds.29452 37226973

